# A multicenter, randomized, double-blind study comparing different FK778 doses (manitimus) with tacrolimus and steroids vs. MMF with tacrolimus and steroids in renal transplantation

**DOI:** 10.1186/1471-2369-13-68

**Published:** 2012-07-26

**Authors:** Zbigniew Wlodarczyk, Yves Vanrenterghem, Bernhard K Krämer, Jean-Paul Squifflet, Marek Ostrowski

**Affiliations:** 1 Klinika Transplantologii, Szpital Uniwersytecki, Bydgoszcz, Poland; 2 Inwendige Geneeskunde-Nefrologie-Nierentransplantatie, Universitaire Ziekenhuizen Gasthuisberg, Leuven, Belgium; 3 V Medizinische Klinik, Universitätsklinikum Mannheim, University of Heidelberg, Mannheim, Germany; 4 Chirurgie Abdominale, Senologique, Endocrine et de Transplantation, Centre Hospitalier Universitaire, Liege, Belgium; 5 Klinika Chirurgii Ogólnej I Transplantacyjnej S.P.S.K. 2, Szczecin, Poland

## Abstract

**Background:**

This multicenter phase II study in renal transplantation compared 3 concentration-controlled ranges of FK778 (manitimus) with mycophenolate mofetil (MMF) both given in combination with tacrolimus and corticosteroids.

**Methods:**

364 patients were randomized to 12-month treatment: high-level FK778 group (H, N = 87) received 4x600mg/day (4 days) followed by 120 mg/day; mid-level FK778 group (M, N = 92) received 3x600mg/day (3 days) followed by 110 mg/day, low-level FK778 group (L, N = 92) received 2x600mg/day (2 days) followed by 100 mg/day, and control group received MMF 1 g/day (MMF, N = 93). After week 6, FK778 doses were adjusted to trough ranges of 75–125 μg/mL (H), 50–100 μg/mL (M) and 25–75 μg/mL (L). Tacrolimus and steroids were administered at the same dose in each of the 4 groups.

**Results:**

Biopsy proven acute rejection (BPAR) at 24 weeks, the primary study endpoint, was comparable in the L (22.8%) and MMF (17.2%) groups but higher in the H (34.5%) and M (29.3%) groups. BPAR at 12 months was comparable in the L (23.9%) and MMF (19.4%) groups but higher in the H (34.5%) and M (31.5%) groups. Graft and patient survival were lowest in the H group and renal function was poorest in the H and M groups. Premature study withdrawal was highest in the H group.

**Conclusions:**

Efficacy was similar between the low-level FK778 and MMF groups. Increased FK778 exposure was poorly tolerated and did not improve efficacy.

## Background

FK778 (manitimus) is a malononitrilamide (MNA) derived from the active leflunomide metabolite teriflunomide which has been found to prevent acute allograft rejection in multiple experimental transplantation models in rodents, dogs, and non-human primates [1]. MNAs suppress T-cell mediated immune reactions and directly affect B-cell responsiveness as well as IgM and IgG antibody production *in vitro* and *in vivo*[2]. Of relevance to immunosuppressed allograft recipients, moderate anti-viral activity against cytomegalovirus (CMV) and BK polyoma virus have been shown with leflunomide [3,4]. MNAs have shown synergistic or additive effects when combined with calcineurin inhibitors [5,6]. FK778 inhibits dihydro-orotate dehydrogenase, interrupting *de novo* pyrimidine synthesis, thereby acting on both B cells and T cells beyond the early S phase of the cell cycle, differentially from calcineurin inhibitors [6].

In a proof of concept clinical trial, renal transplant recipients received FK778 combined with tacrolimus and steroids. Results confirmed the immunosuppressive activity of this combination regimen [7]. In that study, patients received either high-dose FK778 (600 mg twice daily for the first 2 days followed by 150 mg/day) in combination with tacrolimus and steroids, low-dose FK778 (600 mg/day on day 1 followed by 75 mg/day) in combination with tacrolimus and steroids, or a combination of placebo, tacrolimus and steroids. Tacrolimus at an initial dose of 0.2 mg/kg was administered in each group and maintenance tacrolimus trough levels were set at 5-15 ng/ml. Results showed comparable acute rejection rates at 16 weeks in the high (26.5%) and low (25.9%) FK778 groups which were lower than rejection rates in the placebo group (39.1%). The dosing schedule of FK778 used in the proof of concept study yielded FK778 levels < 100 ng/mL in many patients. However in a subgroup of patients in which target levels of FK778 were reached by week 2, acute rejection incidence dropped to 7.7% in the high group but remained similar at 27.1% in the low FK778 group. Graft survival at week 16 was comparable in the high and low FK778 groups.

This study was conducted to evaluate the safety and efficacy of three different regimens of FK778 using concentration controlled dosing over a 12-month period. FK778 was combined with tacrolimus and steroids in all 3 regimens. The previous dose finding study indicated that FK778 plasma concentrations > 100 μg/mL may be most efficacious at preventing rejection during the first 2 weeks after transplantation. At the same time, however, those results indicated difficulty achieving recommended FK778 target levels with higher dose FK778 in the early period after transplantation suggesting that a higher loading dose was necessary to increase the plasma concentration. Based on this experience, this study used concentration-controlled dosing and daily doses were adjusted to maintain plasma concentrations within defined ranges. The loading dose of FK778 was increased to reach set plasma concentrations early after transplantation and target plasma ranges of FK778 were selected to optimize efficacy and tolerability [7]. As a benchmark, a control regimen comprising standard tacrolimus, mycophenolate mofetil (MMF) and steroid regimen was used. The efficacy and safety of FK778 in combination with tacrolimus and steroids was compared to the control regimen.

## Methods

This randomized, double-blind, 4-group, multicenter, phase IIb study was conducted over 12 months in 37 European transplant centers. Patients were adults with end stage kidney disease and low to medium immunological risk (PRA grade ≤ 50%), suitable candidates for renal transplantation or re-transplantation from deceased or living donors including donors meeting expanded donor criteria.

Allocation to treatment was performed in a blinded manner according to a randomization schedule provided by the study sponsor. Randomization took place prior to the administration of the first dose of study medication (FK778 or MMF) and was a 1:1:1:1 randomization stratified by center and donor type (living vs. deceased). Each center was allocated a unique sequence of medication numbers. Investigators remained blinded to the treatment randomization during the conduct of the study.

The immunosuppressive regimens and dosages were as follows:

High level FK778: FK778 loading doses of 600 mg given 4 times on days 1 to 4 followed by initial doses of 120 mg/day. Targeted trough ranges were 150–200 μg/ml until week 4 and 75–125 μg/ml at week 6 and continuing for the remaining study time.

Mid level FK778: FK778 loading doses of 600 mg given 3 times on days 1 to 3 followed by initial doses of 110 mg/day. Targeted trough ranges were 100–150 μg/ml until week 4 and 50–100 μg/ml at week 6 and continuing for the remaining study time.

Low level FK778: FK778 loading doses of 600 mg given 2 times on days 1 and 2 followed by initial doses of 100 mg/day. Targeted trough ranges were 50–100 μg/ml until week 4 and 25–75 μg/ml at week 6 and continuing for the remaining study time.

MMF was administered at a starting dose of 1 g/day given in 2 doses.

Tacrolimus was administered in all 4 treatment groups at a dose of 0.1 mg/kg twice daily. Subsequent doses were adjusted to achieve the following trough ranges: 10–20 ng/mL, days 0 to 30; 5–15 ng/mL, days 31 to 168; and 5–10 ng/mL, days 169 to 365.

Corticosteroids were administered in all 4 treatment groups as a bolus (up to 1000 mg) on day 0 and a 125 mg bolus on day 1. Starting on day 2, oral doses of corticosteroids were tapered until day 168; doses thereafter were at the discretion of the investigator.

First line therapy for acute rejection was corticosteroids according to local practice. Prophylactic antiviral treatment for CMV was required in cases of a CMV positive donor graft and a CMV negative recipient.

The primary endpoint, the incidence of biopsy-proven acute rejection (BPAR) confirmed by local biopsy, was assessed at week 24.

Secondary efficacy and safety endpoints analyzed included: incidence, time to first episode and severity of acute rejection; incidence and time to first episode of corticosteroid-resistant acute rejection; frequency of treatment failure; patient and graft survival; renal function; incidence of adverse events; safety laboratory assessments; CMV and BK polyomavirus load.

Both local and blinded central evaluations of renal biopsies were performed following the Banff classification [8]. Treatment failure was defined as locally evaluated BPAR, graft loss, death, or withdrawal due to an adverse event: the earliest date of any of these events was the date of treatment failure. Graft loss was defined as retransplantation, nephrectomy, death or return to long-term dialysis. The chronic allograft damage index (CADI [9]) was used to measure chronic allograft dysfunction as an indicator of graft outcome. Renal function was measured by serum creatinine concentrations and calculated creatinine clearance (Cockcroft-Gault formula [10]).

The sample size of 70 evaluable patients per treatment group was considered large enough to achieve a power of 80% based on assumed rates of patients with BPAR within 24 weeks of 11%, 18% and 30% in the high, mid and low level FK778 groups, respectively. Assuming a dropout rate of 20%, 85 patients were to be enrolled in each of the 4 treatment groups; thus, a total of at least 340 patients were to be enrolled.

The primary endpoint was analyzed using the one-sided Cochran-Armitage trend test at a 2.5% significance level and stratified by donor type to demonstrate an overall positive correlation of prevention of acute rejection with increasing exposure to FK778. All statistical testing for pair-wise comparisons was two-sided, performed at the 5% level and without adjustment for multiple testing. All analyses of safety and efficacy data were based on the intention-to-treat population (ITT) which included all patients randomized who received at least one dose of FK778 or MMF.

The study was conducted in compliance with the Declaration of Helsinki and Good Clinical Practice guidelines and in accordance with local and national regulatory requirements and laws. All relevant study documents were approved by the Institutional Review Board responsible for the study center. All patients provided signed informed consent and could withdraw from the study at any time.

## Results

The randomized population included 364 patients of which 303 patients (83.2%) completed the study protocol (Table 1). There were more patients prematurely withdrawn from the high level FK778 group (42.5%) than from the mid level FK778 group (35.9%), with a comparable percentage of patients withdrawn from the low level FK778 and MMF groups (22.8% and 23.7%, respectively). The majority of patients (70) were withdrawn due to adverse events which were related to study drug dose (Table 1).

**Table 1:** Disposition of Kidney Transplant Recipients Patients receiving Tacrolimus combined with Three Different Doses of FK778 or with MMF

	**High level FK778**	**Mid level FK778**	**Low level FK778**	**MMF**	**Total**
Intent to treat population (ITT)	87 (100)	92 (100)	92 (100)	93 (100)	364 (100)
Completed study	50 (57.5)	59 (64.1)	71 (77.2)	71 (76.3)	251 (69)
Reasons for study withdrawal:					
Death	0	1 (1.1)	0	0	1 (0.3)
Graft loss/retransplantation	1 (1.1)	1 (1.1)	3 (3.3)	2 (2.2)	7 (1.9)
Adverse event	25 (28.7)	19 (20.7)	14 (15.2)	12 (12.9)	70 (19.2)
Informed consent withdrawn	4 (4.6)	3 (3.3)	2 (2.2)	1 (1.1)	10 (2.7)
Prohibited medication	3 (3.4)	5 (5.4)	0	1 (1.1)	9 (2.5)
Other	4 (4.6)	4 (4.3)	2 (2.2)	6 (6.5)	16 (4.4)

Data are presented as number (percentage).

Patient baseline characteristics were well balanced across groups with the exception of a slightly lower proportion of male recipients and donors in the high level FK778 group (Table 2). Male Caucasian patients predominated, mean age was 47, and all patients had a PRA < 50% and were ABO compatible to their donors. Of the patients with a recorded viral status at baseline, more than half were CMV-positive and approximately 50% were EBV-positive. The most common underlying pathologies leading to chronic renal failure were glomerulonephritis, polycystic disease, uropathy, and nephrosclerosis.

**Table 2:** Demographics and Transplantation Information

	**High level FK778**	**Mid level FK778**	**Low level FK77**	**MMF**
	**N = 87**	**N = 92**	**N = 92**	**N = 93**
Age, mean (SD), y	47.1 (11.8)	48.2 (11.9)	44.9 (12)	46.8 (12.7)
Male, %	56.3	67.4	64.1	72
Caucasian, %	96.6	94.6	96.7	95.7
Viral status, n (%):				
HBV-positive	5 (5.7)	3 (3.3)	6 (6.5)	5 (5.4)
CMV-positive ^a^	58 (66.7)	57 (62)	55 (59.8)	65 (69.9)
HCV-positive ^a^	3 (3.4)	6 (6.5)	3 (3.3)	3 (3.2)
EBV-positive ^a^	47 (54)	45 (48.9)	48 (52.2)	53 (57)
CMV status: donor +/recipient-, n (%)	14 (18.2)	9 (11)	14 (16.9)	11 (12.6)
First renal transplant, n (%)	82 (94.3)	85 (92.4)	82 (89.1)	87 (93.5)
Deceased donor organ, n (%)	82 (94.3)	90 (97.8)	85 (92.4)	85 (91.4)
Cold ischemia time,	15.9 (6.5)	17.2 (6.3)	16.5 (6.7)	17.1 (7.2)
mean (SD), h				
Donor age, mean (SD), y	47.2 (11.2)	44.7 (13.7)	42.9 (13)	43.5 (13.7)
Male organ donor, n (%)	42 (48.3)	57 (62)	58 (63)	61 (65.6)
Mean total HLA mismatch	2.7	2.8	2.5	2.6

^a^ Data on viral status are missing for some patients.

**Figure 1:**
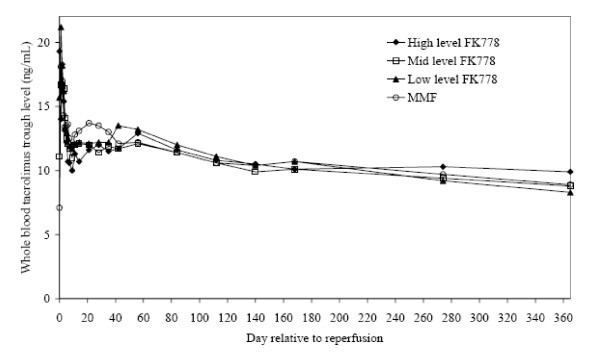
**Mean Tacrolimus Trough Levels during the Study presented for Patients included in the Intent to Treat Population.** With the exception of some variability during the first weeks after transplantation, where tacrolimus levels were highest in the MMF and lowest in the high FK778 group, the mean tacrolimus trough levels were generally similar for all treatment groups. Tacrolimus trough levels showed a gradual decrease in all 4 groups throughout the study.

**Figure 2:**
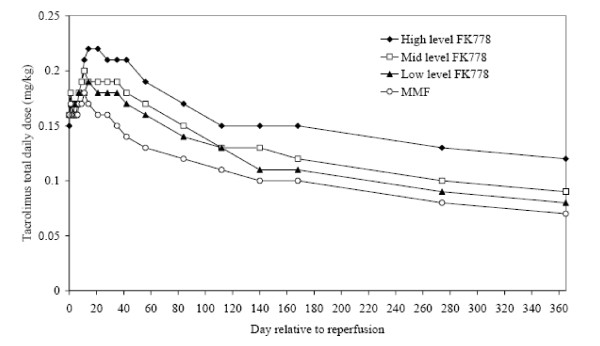
**Mean Total Daily Dose (mg/kg) of Tacrolimus taken by Patients included in the Intent to Treat Population.** The mean tacrolimus dose differed across the treatment groups throughout the study being generally highest in the high level FK778 group and lowest in the MMF group. In all treatment groups tacrolimus doses were highest during the first 3 to 4 weeks of the study and then gradually declined throughout the study.

During the first weeks after transplantation, tacrolimus trough levels were similar across all 4 treatment groups (Figure 1). Higher mean daily doses of tacrolimus were required in the high and mid level FK778 groups, particularly during the first 24 weeks, to maintain targeted tacrolimus trough levels (Figure 2).

**Figure 3:**
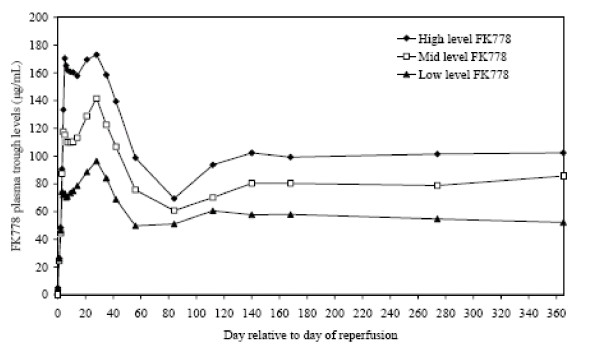
**Mean FK778 Trough Levels during the Study in Patients included in the Intent to Treat Population.** Initial target plasma FK778 trough levels were 150–200 μg/mL (high level FK778), 100 –150 μg/mL (mid level FK778) and 50 –100 μg/mL (low level FK778), and were rapidly achieved in all groups and maintained to week 4 as protocol-specified.

Median FK778 trough levels were within the targeted ranges of 150–200 μg/mL (high level FK778), 100–150 μg/mL (mid level FK778) and 50–100 μg/mL (low level FK778) by days 3, 4, and 5 in all dose groups and maintained to week 4 as protocol-specified (Figure 3). After week 4, daily doses of FK778 were reduced to attain protocol-defined target trough ranges which were maintained until month 12.

All patients in the MMF group received at least 1 g/day of MMF by day 1 and were receiving a mean (SD) daily dose of 1 g (0.12) at month 12.

The incidence of BPAR (local evaluation) at 24 weeks, the study primary endpoint, was significantly higher in the high level FK778 group than in the MMF group (P = 0.010, Fisher’s exact test) (Table 3). The difference in incidence between the high level FK778 and MMF group was 17.3% (95% CI, 4.7 to 29.9%). Differences between the mid and low level FK778 groups and the MMF group were 12.1% and 5.6%, respectively, and did not reach statistical significance. No linear dose–response relationship of FK778 treated groups was found (P = 0.954; Cochran-Armitage trend test stratified by donor type).

**Table 3:** Results from the Intent to Treat Population for Primary and Secondary Efficacy Variables

	**High level FK778**	**Mid level FK778**	**Low level FK778**	**MMF**
	**N = 87**	**N = 92**	**N = 92**	**N = 93**
***at week 24***				
BPAR (local evaluation), n (%)	30 (34.5) ^a^	27 (29.3)	21 (22.8)	16 (17.2) ^a^
***at month 12***				
Acute rejection	35 (40.2)	32 (34.8)	32 (34.8)	21 (22.6)
BPAR (local evaluation), n (%)	30 (34.5)	29 (31.5)	22 (23.9)	18 (19.4)
Treatment outcome:				
Steroid resistant	15 (17.2) ^b^	11 (12)	10 (10.9)	4 (4.3) ^b^
Steroid sensitive	16 (18.4)	19 (20.7)	13 (14.1)	14 (15.1)
Histological grade:				
Mild (Banff I)	9 (10.3)	21 (22.8)	9 (9.8)	6 (6.5)
Moderate (Banff II)	20 (23)	8 (8.7)	13 (14.1)	12 (12.9)
Severe (Banff III)	1 (1.1)	0	0	0
Difference (CI) in BPAR: FK778 vs. MMF	0.151	0.122	0.046	--
	(0.023; 0.279)	(-0.003; 0.246)	(-0.073; 0.164)	
BPAR (central evaluation), n (%)	23 (26.4)	23 (25)	16 (17.4)	16 (17.2)
Treatment failure, n (%)	46 (52.9)	47 (51.1)	34 (37)	29 (31.2)
BPAR (local evaluation)	28 (32.2)	28 (30.4)	21 (22.8)	18 (19.4)
Graft loss	7 (8)	8 (8.7)	4 (4.3)	5 (5.4)
Withdrawal due to AE	9 (10.3)	10 (10.9)	9 (9.8)	5 (5.4)
Withdrawal due to lack of	2 (2.3)	1 (1.1)	0	1 (1.1)
efficacy				
Estimated treatment failure survival rate	0.46	0.46	0.63	0.69
Estimated treatment failure survival rate: FK778 vs. MMF^c^	9.65 (P = 0.002)	5.66 (P = 0.017)	0.54 (P = 0.46)	--

*BPAR* = biopsy proven acute rejection. *CI* = confidence interval. *AE* = adverse event.

^a^ P = 0.010, Fisher’s exact test: high level FK778 vs. MMF. ^b^ P = 0.007, Fisher’s exact test: high level FK778 vs. MMF. ^c^ Chi-square test.

BPAR treatment outcomes may be reported more than once in the same patient.

Treatment failure was the first occurrence of any of these events.

The incidence of BPAR (local evaluation) at 12 months was highest in the high and mid level FK778 groups (Table 3). While the incidence of corticosteroid resistant BPAR was comparable for the FK778 treatment groups, it was lower in the MMF group. The difference in incidence was significantly greater in the high level FK778 group compared with the MMF group (P = 0.007, Fisher’s exact test). More mild (Banff I) rejections were reported in the mid level FK778 group than in the other groups. One severe (Banff III) rejection was reported in the high level FK778 group.

Results of central biopsy evaluation of rejection showed lower incidences of acute rejection in all treatment groups in comparison to biopsy results of local evaluation (Table 3).

**Figure 4:**
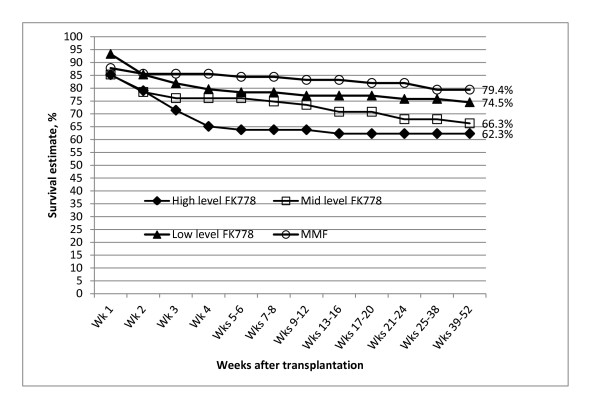
**Estimated Rate of Patients Free from Local Biopsy-proven Acute Rejection (Kaplan-Meier Method).** Differences in the estimated rate of patients free from biopsy proven acute rejection at week 24 between the high level FK778 (62.3%) vs. MMF (82%) groups were significant (P = 0.014, Chi-square test). Differences between the two treatment groups were also significant at month 12 when estimated rates were 62.3% in the high level FK778 vs. 79.4% in the MMF group (P = 0.023, Chi-square test).

Kaplan-Meier survival estimates for freedom from BPAR (local evaluation) at 12 months were lower for the high and mid level FK778 groups than for the low level and MMF groups but comparable between low level FK778 and MMF (Figure 4).

The incidence of treatment failure at 12 months was highest in the high and mid level FK778 groups but comparable between the low level and MMF groups (Table 3). The primary contributor to treatment failure was BPAR in all groups. Kaplan-Meier estimated survival rates from treatment failure at 12 months were lowest in the high and mid level FK778 groups (Table 3).

CADI scores did not indicate a protective effect of FK778 against chronic allograft nephropathy. Renal function, as measured by calculated creatinine clearance, was better in the MMF group compared to the high and mid level FK778 groups. The difference in median values between the low level FK778 and MMF groups was minimal.

One patient in the mid level FK778 group died during the study (death on day 4 due to cardiac arrest) and nine patients died after premature study withdrawal (six patients in the high and two in the low level FK778 groups and one patient in the MMF group). There were no statistically significant differences in patient and graft survival estimates across the treatment groups. Patient survival at 12 months (Kaplan Meier method) was lower for the high (88.4%) compared to the mid (99%) and low (98%) level FK778 groups and the MMF group (99%).

The incidence of adverse events and serious adverse events during the study was generally comparable across groups. At least one adverse event was reported in the majority of patients and a causally-related adverse event in approximately 75% of patients in each treatment group. Adverse events leading to withdrawal were highest in the high and mid level FK778 groups (Table 1). Across all treatment groups, the most common adverse events leading to premature study withdrawal were leukopenia (1–3.4%) and graft dysfunction (0–4.3%).

The most frequently reported adverse event regardless of relationship to study medication was anemia (Table 4). Adverse events associated with a significant difference across treatment groups were thrombocytopenia, chest pain, and hypoalbuminemia (P < 0.05, Fisher’s exact test) (Table 4). The incidence of leucopenia, diarrhea, constipation, insomnia and hyperlipidemia was lower in all FK778 groups compared to the MMF group.

**Table 4:** Most Frequently Reported Adverse Events

	**High level FK778**	**Mid level FK778**	**Low level FK77**	**MMF**
	**N = 87**	**N = 92**	**N = 92**	**N = 93**
Anemia	37 (42.5)	42 (45.7)	36 (39.1)	35 (37.6)
Leukopenia	7 (8)	11 (12)	6 (6.5)	13 (14)
Thrombocytopenia^a^	9 (10.3)	4 (4.3)	11 (12)	2 (2.2)
Diarrhea	14 (16.1)	20 (21.7)	16 (17.4)	23 (24.7)
Constipation	15 (17.2)	8 (8.7)	7 (7.6)	17 (18.3)
Nausea	9 (10.3)	5 (5.4)	6 (6.5)	9 (9.7)
Vomiting	11 (12.6)	12 (13)	7 (7.6)	11 (11.8)
Peripheral edema	13 (14.9)	17 (18.5)	15 (16.3)	12 (12.9)
Chest pain^b^	9 (10.3)	2 (2.2)	2 (2.2)	2 (2.2)
Bacterial urinary tract infection	19 (21.8)	29 (31.5)	28 (30.4)	27 (29)
Cytomegalovirus infection	4 (4.6)	5 (5.4)	9 (9.8)	10 (10.8)
Hypocalcaemia	13 (14.9)	13 (14.1)	12 (13)	9 (9.7)
Hyperglycemia	11 (12.6)	15 (16.3)	10 (10.9)	10 (10.8)
Hyperlipidemia	5 (5.7)	4 (4.3)	2 (2.2)	11 (11.8)
Hypokalemia	15 (17.2)	10 (10.9)	12 (13)	7 (7.5)
Hypoalbuminemia^c^	5 (5.7)	0	1 (1.1)	0
Hyperuricemia	9 (10.3)	21 (22.8)	20 (21.7)	12 (12.9)
Headache	11 (12.6)	11 (12)	9 (9.8)	4 (4.3)
Tremor	9 (10.3)	12 (13)	9 (9.8)	14 (15.1)
Insomnia	4 (4.6)	3 (3.3)	4 (4.3)	10 (10.8)
Hypertension	20 (23)	16 (17.4)	20 (21.7)	25 (26.9)

Data shown are for the ITT and presented as number (%). Most frequently reported adverse events occurring in ≥10% of patients in any group or occurring with a significant difference between groups. Excluded are renal- and allograft-related adverse events.

^a^ P = 0.02, Fisher’s exact test: high level FK778 vs. *MMF*; ^b^ P = 0.03, Fisher’s exact test: high level FK778 vs. *MMF*; ^c^ P = 0.03, Fisher’s exact test: high level FK778 vs. *MMF*.

The incidence of CMV infections was 4.6% in the high level FK778 group with a two-fold higher incidence in the low level FK778 and MMF groups (9.8% and 10.8%, respectively). The plasma CMV DNA load and the plasma and urine polyomavirus BK DNA load were comparable for all treatment groups with similar use of antiviral medication reported across groups (36–41.3%). A malignancy was diagnosed during the study in 5 patients: 3 patients in the high level FK778 group (adenocarcinoma [not further specified], bladder neoplasm, and malignant ascites), 1 patient in the mid level FK778 group (Kaposi’s sarcoma), and 1 patient in the MMF group (malignant neoplasm of the tongue [histology not specified]).

Mean total cholesterol and triglyceride levels were consistently lower in the FK778 groups compared with the MMF group. Overall, the use of lipid-lowering medications was lower in the FK778 groups (26– 28.3%) compared to the MMF group (40%).

## Discussion

The hypothesis that there is a linear dose response relationship between FK778 dose and increasing efficacy could not be proven in this study; increased exposure to FK778 did not result in increased efficacy in terms of acute rejection rates, or less treatment failure or better renal function. In fact, efficacy was lower in the high and mid level FK778 groups compared with the MMF group.

In designing this study, we assumed that rates of BPAR would be lower in the high level FK778 group (i.e., 11%) than in the low level FK778 group (i.e., 30%). Results showed that increasing FK778 exposure did not provide better protection against rejection as the actual rate of BPAR at week 24 in the high level group was three-fold higher than the assumed rate and approximately 10% higher than the actual BPAR rate shown with low level FK778 exposure. Of the three FK778 regimens, low dose FK778 demonstrated similar efficacy to a standard MMF regimen with BPAR rates at 12 months of 23.9% versus 19.4%. The reason for higher incidences of rejection with FK778, especially at the higher dose level, is not clear. One possible explanation is a differential effect on different T-cell subsets with the activity of Tregs being suppressed at higher exposure to FK778.

The administration of loading doses of FK778 resulted in rapid achievement of protocol-defined FK778 trough levels. We used loading doses which were higher than those used in the FK778 proof of concept study [7]. Although these higher loading doses enabled early attainment of trough levels, the rate of BPAR occurring during week 1 was high in both the high and mid level FK778 treatment groups. We also observed that while tacrolimus trough levels were comparable across the FK778 treatment groups, higher daily doses of tacrolimus were required, most notably in the high level FK778 group, to achieve defined trough ranges.

Results of several outcomes at 12 months were similar for the low level FK778 and MMF groups. For example, patient and graft survival rates as well as BPAR incidence by central assessment were similar. The patient and graft survival rates observed in the low level FK778 and MMF groups compare well with a study in which renal transplant recipients received immunosuppressive regimens of tacrolimus, MMF, and corticosteroids or tacrolimus, sirolimus, and corticosteroids: patient survival was greater than 95% and graft survival was greater than 90% [11]. The incidence of treatment failure in our study was numerically lower with MMF than with low level FK778 whereas renal function was found to be comparable.

The premature study withdrawal rates in the low level FK778 and MMF treatment groups were in line with estimated rates used for designing this study. Patient withdrawal rates in the high and mid level FK778 groups were higher than those assumed (20%) and were driven primarily by large numbers of patients who withdrew due to adverse events, notably leukopenia and graft dysfunction. The higher doses of an experimental drug may also have played a role in premature discontinuation in those groups. As observed in previous studies, the most frequently reported adverse events associated with FK778 were blood and lymphatic system disorders; the most frequently reported single adverse event was anemia. The incidences of chest pain, reported significantly more frequently in the high level FK778 group than in the other 3 groups, were investigated by three independent cardiologists who could not find a relationship between FK778 administration and the events.

Previous clinical investigations showed a drop in BK viral load following leflunomide administration [3,4] and we expected to find lower plasma and urine polyomavirus BK DNA load in the FK778 treatment groups. This was not, however, the case. Of clinical interest, FK778 at higher doses might have provided an antiviral effect as shown by the lower incidence of CMV infection in the high level FK778 group compared with the other three treatment groups.

In light of these study results and careful review of the phase II clinical data, no clear benefit of FK778 over current treatment options could be established. In addition, the non-linear pharmacokinetics and the requirement for intensive therapeutic drug monitoring to optimize treatment with FK778 resulted in a decision to discontinue its further clinical development.

## Conclusions

A low dose of FK778 was comparable in terms of efficacy to a standard tacrolimus, MMF, and steroids immunosuppressive regimen. Increased exposure to FK778 did not result in increased efficacy in terms of prevention of acute rejection, treatment failure or improved renal function.

## Competing interests

All authors have received grants for research from Astellas Pharma Europe Ltd. Z. Wlodarczyk, Y. Vanrenterghem. J.P. Squifflet have received lecture fees and travel grants from Astellas Pharma Europe Ltd.; B.K. Kraemer has received lecture fees and travel grants from Astellas Pharma Europe Ltd., BMS, Novartis, Roche, Teva, and Wyeth, grant support from Astellas Pharma Europe Ltd. and Novartis, has served on advisory or safety boards for Astellas Pharma Europe Ltd., BMS, Novartis, Teva, and Wyeth, and has participated in clinical trials sponsored by BMS, Novartis, Roche, and Wyeth.

## Authors’ contributions

All named authors participated in the design of the study and in the performance of the research, developing and reviewing the paper, and in the decision to submit the paper. All authors read and approved the final manuscript.

## Pre-publication history

The pre-publication history for this paper can be accessed here:

http://www.biomedcentral.com/1471-2369/13/68/prepub
